# Supplementary material for: Association of Plasma Total Cysteine and Anthropometric Status in 6–30 Months Old Indian Children
Source: Nutrients. 2020 Oct 15;12(10):3146. doi: 10.3390/nu12103146 (PMC7602373; doi:10.3390/nu12103146)
Supplement: Supplementary file 1 [file nutrients-12-03146-s001.pdf]

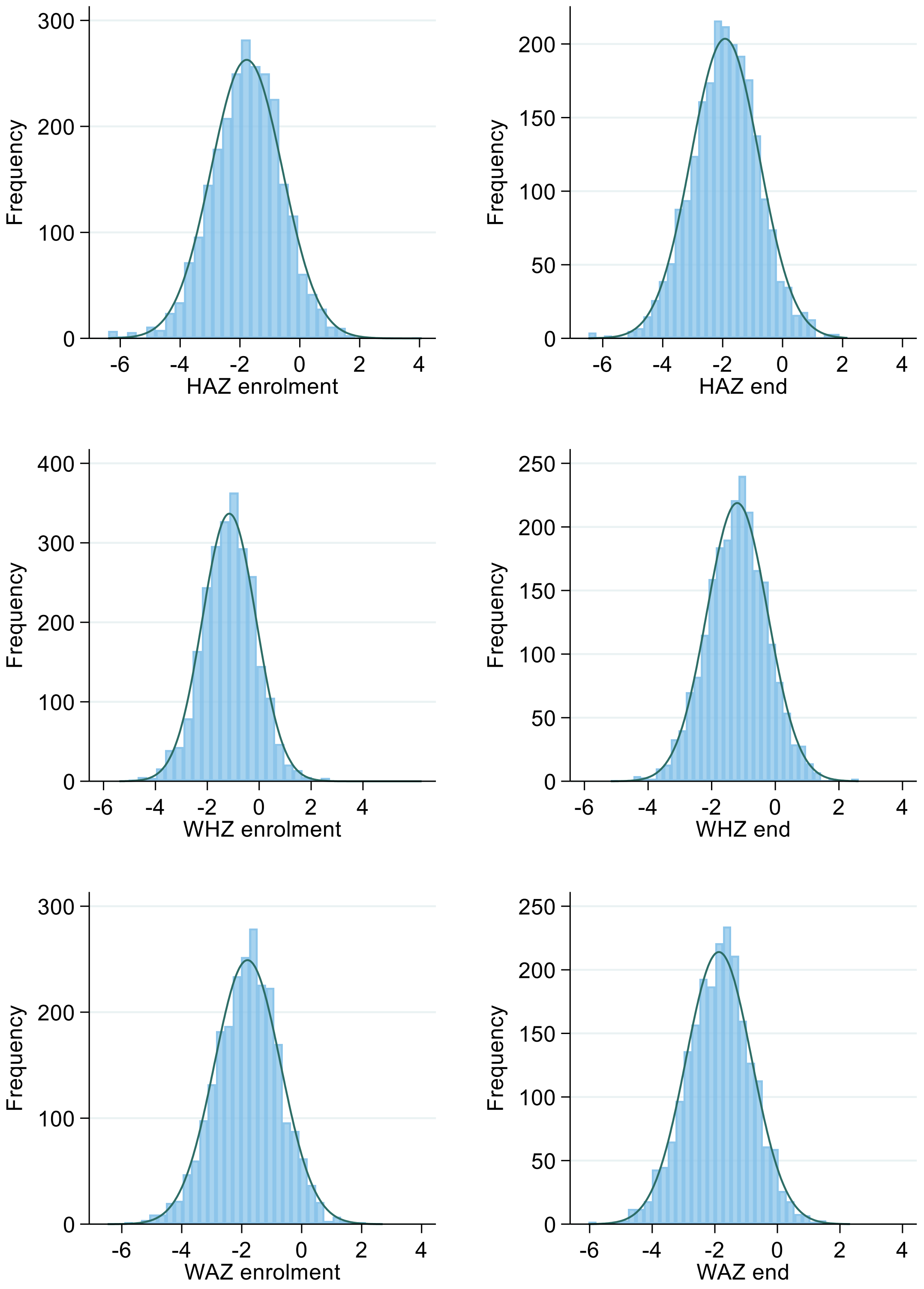

Supplementary Figure S1: Histograms of height-for-age Z-scores (HAZ), weight-for-height Z-scores (WHZ), and weight-for-age Z-scores (WAZ) at enrolment into the study and at the end of the study (4 months after enrolment) of 2,102 children aged 6–30 months living in Delhi, India.
